# Effect of Biochar Type, Concentration and Washing Conditions on the Germination Parameters of Three Model Crops

**DOI:** 10.3390/plants12122235

**Published:** 2023-06-07

**Authors:** Pablo Carril, Majid Ghorbani, Stefano Loppi, Silvia Celletti

**Affiliations:** 1 Department of Life Sciences, University of Siena, 53100 Siena, Italy; 2 BAT Center—Interuniversity Center for Studies on Bioinspired Agro-Environmental Technology, University of Naples ‘Federico II’, 80055 Naples, Italy

**Keywords:** germination tests, biochar, basil, lettuce, tomato

## Abstract

Biochar has been recognized as a promising and efficient material for soil amendment. However, its effects on seed germination are variable due to its alkaline pH and/or the presence of phytotoxic substances. In this study, two types of biochar (B1 and B2) were mixed with soil at different concentrations (0%, 5%, 10%, 25%, 50% and 100%, w:w), and both the solid and liquid fractions of these mixtures were tested on the germination of basil, lettuce and tomato seeds. Furthermore, solid fractions subjected to a pre-washing treatment (B1_W_ and B2_W_) were also investigated for their effects on seed germination. Three germination parameters were then measured: seed germination number (GN), radicle length (RL) and germination index (GI). Biochar B2_W_ at 10% increased both RL and GI in basil by 50% and 70%, respectively, while B1 at 25% increased these parameters in tomato by 25%. No effects or negative effects were recorded for lettuce. Liquid fractions (L1 and L2) generally hampered seed germination, suggesting the presence of potentially water-soluble phytotoxic compounds in biochar. These results point to biochar as a suitable component for germination substrates and highlight that germination tests are critical to select the best performing biochar according to the target crop.

## 1. Introduction

Seed germination refers to the process starting with the imbibition of seeds and terminating with the emergence of the radicle [1]. It is one of the most delicate stages in plant development as seeds are easily vulnerable to mortality from drought, granivores and soil-borne pathogens [2]. Seeds are the starting point to initiating a new crop cycle, and their adequate germination critically determines the trajectories of crop success or failure in agricultural systems [3]. Considering that crop varieties are selected based on the quality of their seeds in competitive markets, ensuring successful germination and seedling establishment is a significant step in agricultural production [4]. In this context, adequate plant-growing substrates are essential to provide the optimal conditions for germination. They generally include varying concentrations of commercially available blends of organic and inorganic materials, such as peat moss or vermiculite [5]. However, despite these materials providing appropriate conditions for seed germination (e.g., pore-water retention and aeration), their cost has gradually increased over the years. Furthermore, the use of peat and vermiculite is limited by the expense of mining these materials, as they are both finite resources, and by their scarce availability in regions where they are not naturally present [6,7]. Therefore, alternative functional materials for soil amendment are increasingly needed in order to improve seed germination in a more sustainable way.

In these circumstances, the energy valorization of waste plant biomass (e.g., residues from agricultural and forestry processing, residues from the wood and paper industry or waste from agro-food products intended for human or animal consumption) offers new possibilities to create more sustainable materials which can be reused in agriculture [8]. Biochar is a type of charcoal obtained from the pyrolysis of plant biomass in oxygen-limited conditions and temperatures between 250 °C and 1000 °C [9,10]. This material has gained substantial attention in the agricultural sector due to its potential to reduce the bioavailability of toxic elements in the soil [11,12], to increase carbon sequestration or to increase soil water holding capacity, soil cation exchange capacity and soil organic matter content [13,14]. These positive effects have pointed to biochar as a valuable substrate component for plant production [15].

However, the effects of biochar on seed germination can be variable depending on the plant species analyzed, as well as on the type of the biochar used (i.e., its processing conditions and/or the feedstock biomass employed to produce it) [8]. For example, seed germination studies employing biochars from different plant sources have shown positive effects on the germination of potato (*Solanum tuberosum* L.), sunflower (*Helianthus annuus* L.) and wheat (*Triticum aestivum* L.) [16,17,18,19]. On the other hand, a reduction in seed germination percentage after biochar amendment has been reported for mung bean (*Vigna radiata* L.), subterranean clover (*Trifolium subterraneum* L.) and wheat [19]. These negative effects may be attributed to the existence of phytotoxic substances in biochar, e.g., polycyclic aromatic hydrocarbons (PAHs), phenolics, benzoate and cinnamate derivatives, which can negatively affect seed germination, as shown for black maize (*Zea mais* L.), mustard (*Brassica nigra* L.) or alfalfa (*Medicago sativa* L.) [20,21,22]. In addition, the alkaline nature of biochar can cause osmotic stress and nutrient imbalance, as high pH can lead to the insolubilization, and thus the unavailability, of important nutrients for plant growth [23]. In relation to this, several studies have proposed different strategies to reduce biochar phytotoxicity. One such strategy foresees a prior washing step with either water or organic solvents. This method has been shown to both remove harmful soluble chemical constituents of biochar [24,25], as well as to reduce its excessive alkalinity [26].

Considering the variable effects of biochar on seed germination, testing this material for soil amendment is critical for its agronomic acceptance, as it should not cause abiotic stress for the germinating seeds. Therefore, germination tests are required in order to investigate biochar’s suitability as a component of plant growing substrate [20,24].

In the present work, soils amended with two biochar types (B1 and B2) derived from sweet chestnut biomass, applied at different concentrations (0%, 5%, 10%, 25%, 50% and 100%, w:w) and subjected to different washing conditions (pre-washed or non-pre-washed), as well as their solid and liquid fractions, were investigated in light of their capacity to improve different germination parameters (germination number, radicle length, and germination index) in three model crops: lettuce (*Lactuca sativa* L.), basil (*Ocimum basilicum* L.) and tomato (*Solanum lycopersicum* L.).

## 2. Results

### 2.1. Germination Tests

According to the 3-way ANOVA analyses (Table 1), GN, RL and GI parameters were differentially influenced by biochar concentration, biochar type and washing pre-treatment. Concerning GN in basil seeds, differences were dependent on biochar concentration, washing treatment and their combined interaction (*p* < 0.0001). In the case of tomato, differences in GN were driven by the interaction of both biochar concentration and type as well as by the interaction of the three factors (*p* < 0.005). 

On the other hand, significant differences in RL and GI across the three plant species were consistently driven by biochar concentration (*p* < 0.0001 for basil and tomato, *p* < 0.001 for lettuce), by the interaction of biochar concentration and washing treatment (*p* < 0.0001) as well as by the interaction between the three factors (*p* < 0.0001 for basil and lettuce; *p* < 0.05 for tomato). Further differences in these two parameters were also dependent on biochar type in both lettuce and tomato (*p* < 0.0001), while those driven by washing treatment affected RL (*p* < 0.05, for basil; *p* < 0.0001 for lettuce; *p* < 0.005 for tomato) and GI only in the case of tomato (*p* < 0.0001).

**Figure 1:**
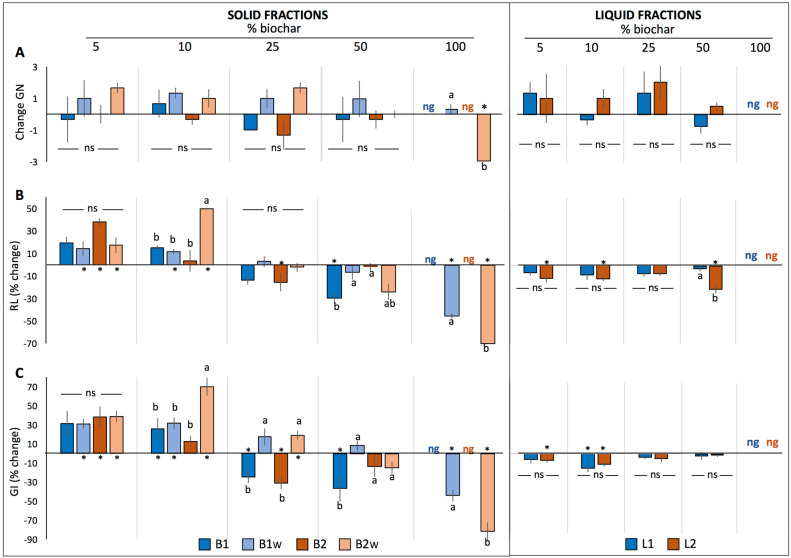
Change in the number of germinated seeds (GN) (**A**), % change in radicle length (RL) (**B**) and % change in germination index (GI) (**C**) of basil seeds (mean ± SE) germinated in the solid fractions (B1, B1_W_, B2 and B2_W_) or in the liquid fractions (L1 and L2) relative to control (0% biochar). Asterisks (*) indicate significant changes relative to control, while different letters indicate significant differences among treatments within each biochar concentration. Abbreviations: ns: not significant; ng: not germinated.

**Figure 2:**
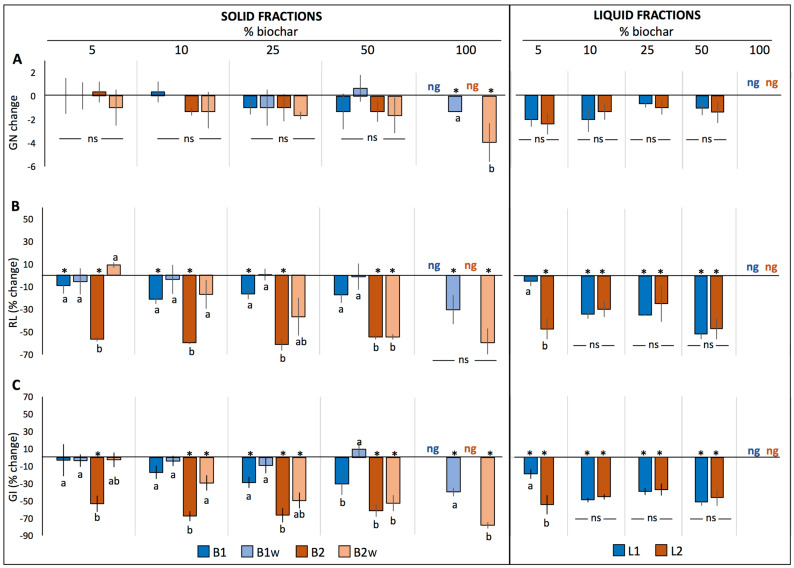
Change in the number of germinated seeds (GN) (**A**), % change in radicle length (RL) (**B**) and % change in germination index (GI) (**C**) of lettuce seeds (mean ± SE) germinated in the solid fractions (B1, B1_W_, B2 and B2_W_) or in the liquid fractions (L1 and L2) relative to control (0% biochar). Asterisks (*) indicate significant changes relative to control, while different letters indicate significant differences among treatments within each biochar concentration. Abbreviations: ns: not significant; ng: not germinated.

**Figure 3:**
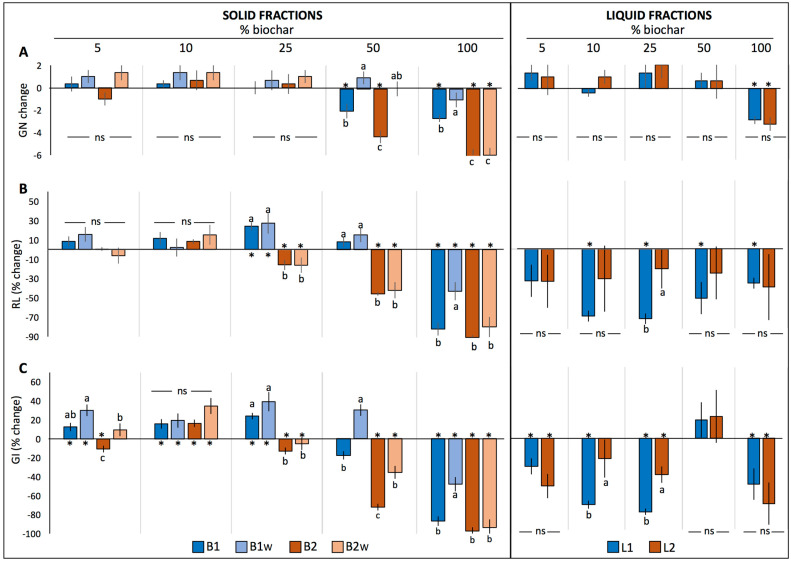
Change in the number of germinated seeds (GN) (**A**), % change in radicle length (RL) (**B**) and % change in germination index (GI) (**C**) of tomato seeds (mean ± SE) germinated in the solid fractions (B1, B1_W_, B2 and B2_W_) or in the liquid fractions (L1 and L2) relative to control (0% biochar). Asterisks (*) indicate significant changes relative to control, while different letters indicate significant differences among treatments within each biochar concentration. Abbreviations: ns: not significant.

Significant interactions resulting from the three-way ANOVA were decomposed into a one-way model to determine the differences between treatments in GN, RL and GI within each biochar concentration (Figure 1, Figure 2 and Figure 3). In general terms, germination tests in both solid (B1, B1_W_, B2 and B2_W_) and liquid fractions (L1 and L2) showed that biochar did not significantly change GN in the three tested species compared to the control (Figure 1A, Figure 2A and Figure 3A), and no differences between treatments were found except for the 100% treatments, where both basil and lettuce seeds did not germinate at all neither in B1 nor in B2. However, in the case of tomato, significant differences were detected at both 50% and 100% concentration, where GN remained unchanged only in B1_W_, while it decreased significantly in the rest of the treatments. Nevertheless, substantial differences were found for both RL and GI relative to the plant species analyzed, as described in the following sections.

#### 2.1.1. Basil

Considering the germination tests in the solid fractions, both RL and GI generally increased or remained unchanged in both 5% and 10% biochar treatments compared to control (Figure 1B,C). Notably, in the 10% concentration, B2_W_ showed the most remarkable effect in both parameters compared to the other treatments, increasing RL and GI by 50% and 70%, respectively. Regarding the 25% treatments, RL significantly decreased only in B2 and remained unaffected in the rest. On the other hand, GI increased in both B1_W_ 25% and B2_W_ 25%, with this increase being significant only in the latter, whereas it decreased in their respective non-washed counterparts. Biochar treatments above 25% concentrations had either no effect or a negative effect on both tested parameters. 

Germination tests carried out in the liquid fractions showed that both RL and GI were either not affected or negatively affected in all concentrations tested. Specifically, significant decreases were observed in L2 at 5%, 10% and 50% (between 10 and 20% decrease in RL), as well in both L2 at 5% and 10% (7.5 and 12% decrease in GI) and in L1 10% (16% decrease in GI).

#### 2.1.2. Lettuce

Considering the germination tests in the solid fractions, both RL and GI were either not affected or negatively affected by biochar treatments compared to control (Figure 2B,C). In particular, B2 showed the most marked negative effect in all concentrations compared to the other treatments, consistently decreasing both RL and GI from 50% to 60% (*p* < 0.05). However, the observed decrease in both parameters was more attenuated when both types of biochar were washed (B1_W_ and B2_W_) and this effect was particularly visible for B1_W_, where both RL and GI did not change compared to control. Germination tests carried out in the liquid fractions showed that both RL and GI significantly decreased independently of the treatment, with the exception of L1 5%, which was similar to control.

#### 2.1.3. Tomato

Considering the germination tests in the solid fractions, both RL and GI were differently affected by biochar treatments relative to control (Figure 3B,C). On one hand, RL was not affected up to the 10% treatments and it increased significantly only in B1 25% and B1_W_ 25% (*p* < 0.05). On the other hand, GI increased significantly in both B1 and B1_W_ up to the 25% concentration as well as in B2 10% and B2_W_ 10%.

Similar to the other plant species, all liquid fractions tested showed either a not significant or a negative effect on both RL and GI.

### 2.2. Effects of Biochar Addition on pH and EC

**Figure 4:**
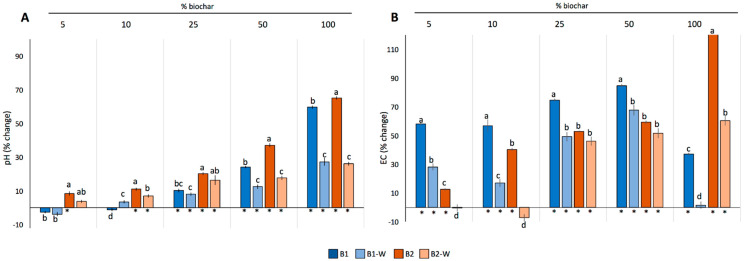
Changes in pH (**A**) and EC (**B**) in the different soil–biochar mixtures. Asterisks (*) indicate significant changes of both pH and EC relative to control, while different letters indicate significant differences among treatments in each soil–biochar mixture.

Biochar treatments generally increased the pH of the substrate compared to control, and this increase was more pronounced at higher biochar concentrations (Figure 4A, Supplementary Table S1).

In the case of B1 and B1_W_, they both increased pH significantly compared to the control when applied at 25% concentrations or higher (*p* < 0.05), while B2 and B2_W_ increased it from the 5% and 10% treatments, respectively. The pre-washing step attenuated this pH-increasing effect in B1-containing mixtures, especially in the 50% and 100% concentrations (from pH 7.5 to 7 and from pH 9.5 to 7.8, respectively). Similar results were observed for B2 mixtures at the same concentrations (from pH 8 to 7 and from pH 10 to 8, respectively). Opposite trends between B1 and B2 were observed in the 10% concentration, where the pre-washing step decreased pH in B2, and increased it in B1. Soil EC significantly changed in all biochar treatments compared to control (*p* < 0.05) (Figure 4B, Supplementary Table S1). It increased in all treatments, especially in non-washed biochars: B1 increased EC significantly more compared to B2 at all concentrations applied, except in the 100% concentration. Both washed biochars decreased soil EC compared to their non-washed counterparts, and this decrease was significant across all concentrations in the case of B1 and in the 5, 10 and 100% concentrations in the case of B2.

## 3. Discussion

In this study, the effects of two biochar types applied at different concentrations and under different washing conditions were investigated on three germination parameters of three model crops. In general terms, the effects of biochar addition led to qualitative, rather than quantitative, changes in the germination of the considered plant species. Specifically, it significantly affected both RL and GI, but not GN. Not significant changes in GN suggest that biochar did not affect seed endosperm weakening and the emergence of radicles (germination in the strict sense); this is consistent with previous works on maize [27,28], European beech, Turkey oak [29] and different herbaceous perennial species [30]. Nevertheless, biochar alone (100% treatments) clearly compromised GN. This result may be attributed to the alkalinity (pH 9.5 and 9.8 for B1 and B2, respectively) and/or the excessive content of eventual inorganic (i.e., salts) and organic contaminants (e.g., organic acids, phenols, aldehydes or PAHs) in the biochar not subjected to a washing pre-treatment, hence leading to GN inhibition [24,31]. However, a reduced phytotoxic effect was observed in some cases when B1 100% was subjected to a washing treatment, suggesting that washing this biochar could reduce the levels of both pH and eventual contaminants.

As for qualitative changes, solid biochar fractions had different effects on RL and GI depending on the plant species. In particular, both B1 and B1_W_ (at 25% *w*:*w*) and B2_W_ (at 10% *w*:*w*) showed the best effects for tomato and basil seeds, respectively, significantly increasing both RL and GI. These positive effects could be linked to changes in soil pH and EC when biochar is applied at specific concentrations. The best performing biochar mixtures in basil and tomato (B1 25% and B2_W_ 10%, respectively) increased the pH of the substrate to a similar extent (from pH 6–6.2 to 6.5–6.6). Increases in pH due to the alkaline nature of biochar have been related to higher nitrification in the soil [32,33] as well as to a decrease in P adsorption by soil particles [34,35]. Hence, germinating seeds may benefit from an extra supply of these nutrients in the presence of biochar by means of the observed pH increases in the substrate. Moreover, the observed increases in soil pH driven by specific biochar concentrations could also have limited the mobility of toxic elements in the soil, and thus their availability for plants [11].

Changes in EC may also have impacted germination as most biochars contain high amounts of soluble salts (e.g., Na^+^ and Cl^−^), and thus a higher EC than most agricultural soils [36]. This could negatively affect the availability of important soluble nutrients (e.g., NO_3_^−^, K^+^, and Ca^2+^), leading to nutrient imbalance and decrease in plant osmotic potential [37,38]. However, both the negligible EC decrease in B2_W_ (from 162 to 150 µS cm^−1^) as well as the drastic increase B1 (from 396 to 691 µS cm^−1^) did not seem to affect germination. Different effects were in turn observed for lettuce, which has been previously described as a sensitive species to increases in pH and EC [39,40]. Nevertheless, this species was not negatively impacted by low to medium doses (5% to 25%) of B1_W_ as well as by low doses of B2_W_ (5%). In this regard, the improvement in germination observed in these washed mixtures may be related to the fact that both pH and EC increases were attenuated by the washing treatment.

Notably, our results show that the negative impacts caused by biochar may be related to potentially water-soluble phytotoxic compounds present in the liquid fractions of the soil–biochar mixtures. This was particularly evident for basil, where washing B2 significantly improved its effects on RL and GI compared to its non-washed counterpart, suggesting that pre-washing this biochar may be particularly effective in reducing its phytotoxicity when used at concentrations higher than 5% (*w*:*w*). These observations are consistent with previous results, showing that germination improved when biochars were subjected to a prior washing treatment using either water or organic solvents (e.g., methanol, hexane, dichloromethane or toluene) [20,24,26]. In agreement with this, germination tests carried out using biochar liquid fractions showed negative effects on the majority of the analyzed parameters, as previously observed in the germination of maize, rice [41], clover and mung bean seeds [19]. The slowdown of plant growth could be related due to the presence of toxic metals, VOCs and PAHs and/or the presence of free radicals in biochar [26,42]. However, to the best of our knowledge, it remains unknown which compounds, their concentrations and/or interactions are mainly responsible for the observed negative effects on the germination of the species considered in this study [27,43,44,45]. In this regard, considering that many VOCs and PAHs, formed during the pyrolysis process, represent important groups of phytotoxic compounds in biochar [26,46], further studies employing gas chromatography and spectrometry techniques will significantly contribute to identify both the nature and the amount of both potentially beneficial (e.g., nutrients) and/or harmful (e.g., heavy metals, PAHs, VOCs) compounds that may be removed during the biochar washing process. In addition, further germination tests will be necessary in order to have deeper insights on their dose–response effect on plants.

The present results suggest that soil amendment with biochars B1 or B2 can differentially improve the quality of germination in both basil and tomato, but not in lettuce. Importantly, special attention has to be taken when choosing both the type and the concentration of the biochar used in relation to the plant species analyzed. In specific cases, as observed for B2 effects on basil, a pre-washing step of biochar should also be considered to reduce its potential phytotoxic effects on germination.

## 4. Materials and Methods

### 4.1. Soil–Biochar Mixtures

Two different types of biochars obtained from sweet chestnut (*Castanea sativa* Mill.) (referred to as B1 and B2, respectively) were kindly provided by BioDea^©^ (Arezzo, Italy) The two biochars originated from the same feedstock but were the result of different operating conditions. Biochars were ground with mortar and pestle until a fine powder. Subsequently, 8 g of six different mixtures were prepared in triplicate by mixing a commercial plant growing soil (VigorPlant Srl, Lodi, Italy) with different concentrations of either B1 or B2: 0%, 5%, 10%, 25%, 50% and 100% (*w*/*w*). These mixtures gave rise to the following treatments: B1 0% (control), B1 5%, B1 10%, B1 25%, B1 50%, B1 100%; and B2 0% (control), B2 5%, B2 10%, B2 25%, B2 50%, B2 100%, which were used for subsequent germination tests. The physicochemical characteristics of both biochars and the soil used are listed in Table 2.

### 4.2. Germination Test

Soil–biochar mixtures were moistened by adding deionized water (dH_2_O) at a ratio of 1:2 (g:mL), shaken on a vortex (Techno Kartell TK3S, Noviglio, MI, Italy) for 5 s at maximum speed and spread on plastic Petri dishes (11 cm diameter) to uniformly cover the entire surface. Seeds of lettuce (*Lactuca sativa* L., cv. “Verde degli Ortolani”), basil (*Ocimum basilicum* L., cv. “Riviera Ligure”) and tomato (*Solanum lycopersicum* L., cv. “Principe Borghese”) were chosen as model plant species due to their fast germination rate and sensitivity to toxic substances. Seeds were soaked in dH_2_O under gentle stirring for 2 h and then transferred (10 seeds/Petri dish) on the layer of each soil–biochar mixture previously prepared. Dishes were sealed with parafilm and kept at 24 °C in darkness until the radicle length reached 2 mm (72 h in the case of lettuce and basil, and 96 h in the case of tomato). At this stage, the number of germinated seeds (germination number: GN) was first counted and recorded. Subsequently, seedlings were placed on a black background and their images were captured using a digital camera (Canon EOS 400). Hence, radicle length (RL) was measured using the “Segmented line” tool of the Fiji/ImageJ software, by setting the scale of the image to 109.4 pixels = 1 mm. Both the number of germinated seeds and seedling’s RL measurements were then used to calculate the germination index (GI, %) according to the following formula [48]:

$$GI\left(\%\right)=\left[\frac{\left(G_{t}\times L_{t}\right)}{\left(G_{c}\times L_{c}\right)}\right]\times 100$$

where *G_t_* = mean number of germinated seeds in the treatment; *L_t_* = mean total RL (cm) in the treatment; *G_c_* = mean number of germinated seeds in the control treatment (0% biochar); *L_c_* = mean total RL (cm) in the control (0% biochar). 

### 4.3. Germination Test in the Washed Mixtures and in the Liquid Fractions

Soil–biochar mixtures were washed by adding dH_2_O at a ratio of 1:20 (g:mL) and continuously shaken for 2 h in a rotary shaker (711+, VDRL STIRREL, ASAL S.r.l., Cernusco sul Naviglio, MI, Italy). Subsequently, they were centrifuged at 4000 rpm for 5 min (PK110 centrifuge, Alc International S.r.l., Cologno Monzese, MI, Italy), obtaining a solid and a liquid fraction for each sample. Solid fractions were recovered, air-dried until a constant weight, moistened by adding dH_2_O at a ratio of 1:2 and transferred to new Petri dishes following the procedure described in Section 2.2. These washed mixtures gave rise to the following treatments: B1_W_ 0% (control) B1_W_ 5%, B1_W_ 10%, B1_W_ 25%, B1_W_ 50%, B1_W_ 100%; and B2_W_ 0%, B2_W_ 5%, B2_W_ 10%, B2_W_ 25%, B2_W_ 50%, B2_W_ 100%. The liquid fractions were filtered using filter papers (12 mm diameter, pore size: 11µm, S.r.l Prokeme S.r.l., Calenzano, Italy) and the resulting filtrates were used for wetting a clean filter paper placed into new Petri dishes. These liquid fractions gave rise to the following treatments. L1 0% (control), L1 5%, L1 10%, L1 25%, L1 50%, L1 100%; and L2 0%, L2 5%, L2 10%, L2 25%, L2 50%, L2 100%. Both fractions were used to carry out the seed germination tests for the three plant species as described in Section 2.2.

### 4.4. Measurement of pH and EC

Both pH and electrical conductivity (EC) were measured in the filtered liquid fractions of each sample according to [48] using a pH-meter (edge^®^ HI2002, HANNA Instruments Inc., Woonsocket, RI, USA) and an EC-meter (BASIC 30, EC—meter, Crison Strumenti SpA, Carpi, Italy), respectively.

### 4.5. Statistical Analysis

The data approached a normal distribution (Shapiro–Wilk test, *p* < 0.05). A Student’s *t*-test (*p <* 0.05) was first run to check for statistically significant differences in the measured parameters (GN, RL, GI, pH and EC) between each treatment and the control. Subsequently, each treatment was expressed as ratio to the control and a three-way analysis of variance (ANOVA) was first used to study the effects of biochar type (B1 or B2), biochar concentration (0% to 100%) and washing treatment (pre-washed or non-pre-washed), as well as their interactions, on the parameters measured in the solid fractions. Then, a one-way analysis of variance, followed by Tukey’s post hoc test (*p* < 0.05), was used to find significant differences between treatments within each biochar concentration (in the case of the solid fractions, pH and EC). In the case of the liquid fractions, a Student´s *t*-test (*p* < 0.05) was used to compare between treatments within each biochar concentration.

## 5. Conclusions

Biochar addition to the soil did not affect GN in any of the species tested when seeds were germinated in either the solid or in the liquid fractions of the prepared soil–biochar mixtures, with exception of the 100% biochar concentration. On the other hand, the interaction between biochar type, concentration and washing pre-treatment differentially affected both RL and GI according to the species analyzed. Basil and tomato seeds significantly increased both parameters when germinated in soils amended with B2_W_ 10% or B1 25%, respectively. Notably, pre-washed B2 significantly improved both RL and GI in basil compared to its non-washed counterpart, suggesting that a pre-washing step may be recommended to reduce its potential phytotoxic effects for this specific species. In this regard, the phytotoxic effects of biochar could be attributed to the presence of phytotoxic, water-soluble compounds, as confirmed by the negative effects of the liquid fractions on both RL and GI in all plant species. These results suggest that the biochars employed in this study hold a great potential to improve seed germination, which is a critical agronomic trait affecting crop quality and productivity. Furthermore, the different effects of biochar type, concentration and washing conditions in each plant species suggest that an exhaustive evaluation of these variables through germination tests is critical to select the best performing biochar mixtures prior to their use for plant propagation purposes.

In the quest for alternative and more sustainable soil amendments to improve seed germination in agricultural plants, recycled materials such as biochar could significantly contribute to improving the germination of target crops while having lower environmental and economic costs compared to other finite and more expensive materials, such as peat or vermiculite.

## Figures and Tables

**Table 1:** Results from the three-way ANOVA analysis (*p* values) showing both the main and combined effects of biochar concentration (C), type (T) and washing treatment (W) on GN, RL and GI.

	*O. basilicum*			*L. sativa*			*S. lycopersicum*		
	GN	RL	GI	GN	RL	GI	GN	RL	GI
C	1.81^−12^ ***	2^−16^ ***	2^−16^ ***	ns	4.8^−3^ **	2.25^−3^ **	ns	2^−16^ ***	2^−16^ ***
T	ns	ns	ns	ns	2^−16^ ***	2.5^−16^ ***	ns	2^−16^ ***	2^−16^ ***
W	7.19^−9^ ***	2.47^−2^ *	ns	ns	7.1^−4^ ***	ns	ns	3.5^−3^ **	1.4^−15^ ***
C*T	ns	3.68^−2^ *	0.012 *	ns	9.4^−4^ ***	ns	4.21^−3^ **	2^−16^ ***	9.2^−15^ ***
C*W	2.04^−5^ ***	3.7^−16^ ***	2^−16^ ***	ns	2^−16^ ***	9.2^−14^ ***	ns	4.5^−4^ ***	2.3^−4^ ***
T*W	ns	ns	ns	ns	ns	ns	ns	ns	ns
C*T*W	ns	4.95^−6^ ***	7.26^−5^ ***	ns	6^−6^ ***	4.6^−4^***	7.21^−3^ **	0.016 *	0.02 *

* *p* < 0.05; ** *p* < 0.005: *** *p* < 0.0001.

**Table 2:** Physicochemical characteristics of biochar B1, biochar B2 and soil used in this study.

Characteristics	B1	B2	Soil
Particle diameter (mm)	<2	<2	
Particle diameter (ground, mm)	<0.5	<0.5	
Total nitrogen (%)	<0.4	<0.4	
Total potassium (mg kg^−1^)	3020	3020	
Total phosphorus (mg kg^−1^)	340	340	
Total calcium (mg kg^−1^)	9920	9920	
Total magnesium (mg kg^−1^)	852	852	
Total sodium (mg kg^−1^)	291	291	
Carbon from carbonate (%)	<0.1	<0.1	
Total carbon (%)	68.7	68.7	
Water holding capacity (%)	23.5	23.5	
Has content (%)	4.6	4.6	
Carbon/hydrogen ratio	0.2	0.2	
Available phosphorus (mg kg^−1^, this study *)	50 ± 4	34 ± 2	
EC (µS cm^−1^, this study **)	552 ± 5.7	999 ± 3.2	400 ± 3.3.
pH (this study **)	9.55 ± 0.03	9.88 ± 0.03	5.98 ± 0.07
CEC (meq 100 kg^−1^, this study ***)	90 ± 6	115 ± 3	553 ± 38

* determined according to [47]. ** EC (electrical conductivity) and pH, determined according to [48]. *** CEC (cation exchange capacity), determined according to [49].

## Data Availability

The data presented in this study are available on request from the corresponding author.

## Supplementary Materials

The following supporting information can be downloaded at: https://www.mdpi.com/article/10.3390/plants12122235/s1, Table S1: Values of pH and EC (mean ± SE) of the different soil-biochar mixtures.
