# Supplementary material for: Effect of Biochar Type, Concentration and Washing Conditions on the Germination Parameters of Three Model Crops
Source: Plants (Basel). 2023 Jun 7;12(12):2235. doi: 10.3390/plants12122235 (PMC10303376; doi:10.3390/plants12122235)
Supplement: Supplementary file 1 [file plants-12-02235-s001.zip › plants-2420080-supplementary.pdf]

Supplementary Table S1. Values of pH and EC (mean ± SE) in the different soil biochar mixtures.

% biochar	pH				EC			
	**B1**	**B1w**	**B2**	**B2w**	**B1**	**B1w**	**B2**	**B2w**
**0%**	6±0.07	6.2±0.1	6±0.07	6.2±0.1	395±3	162±5	395±3	162±5
**5%**	6±0.03	6±0.12	6.5±0.2	6.5±0.03	627±6	206±4	446±6	161±7
**10%**	6±0.08	6.4±0.05	6.7±0.06	6.5±0.05	620±33	189±6	555±6	150±8
**25%**	6.6.±0.1	6.6±0.05	7.19±0.05	7±0.34	691±5	241±5	604±3	236±5
**50%**	7.5±0.03	7±0.14	8.2±0.15	7.5±0.15	731±6	271±6	630±6	245±7
**100%**	9.5±0.02	7.8±0.26	10±0.03	7.7±0.06	542±5	164±5	1005±4	259±5
